# Postoperative Diffusion-Weighted Imaging Hyperintensity Following Combined Photodynamic Diagnosis and Therapy Using 5-Aminolevulinic Acid and Talaporfin Sodium in Malignant Brain Tumors

**DOI:** 10.3390/cancers18152479

**Published:** 2026-08-02

**Authors:** Takumi Inaba, Narushi Sugii, Hidehiro Kohzuki, Shunichiro Miki, Takao Tsurubuchi, Masahide Matsuda, Eiichi Ishikawa

**Affiliations:** 1 Department of Neurosurgery, Mito Medical Center, National Hospital Organization, Ibaraki 311-3193, Ibaraki, Japan; inaba.takumi.sl@ms.hosp.tsukuba.ac.jp; 2 Department of Neurosurgery, Institute of Medicine, University of Tsukuba, Tsukuba 305-8577, Ibaraki, Japan; miki.shunichiro.dx@ms.hosp.tsukuba.ac.jp (S.M.); m-matsuda@md.tsukuba.ac.jp (M.M.); e-ishikawa@md.tsukuba.ac.jp (E.I.); 3 Department of Neurosurgery, Ibaraki Prefectural Central Hospital, Kasama 309-1793, Ibaraki, Japan; h.kohzuki@md.tsukuba.ac.jp (H.K.); t-tsurubuchi@gwe.md.tsukuba.ac.jp (T.T.); 4 Ibaraki Clinical Education and Training Center, Institute of Medicine, University of Tsukuba, Tsukuba 305-8575, Ibaraki, Japan

**Keywords:** 5-aminolevulinic acid, diffusion-weighted imaging, hyperintensity, photodynamic diagnosis, photodynamic therapy, talaporfin sodium

## Abstract

**Background/Objective**: Intraoperative local photodynamic therapy (PDT) using talaporfin sodium (TS) and photodynamic diagnosis (PDD) with 5-aminolevulinic acid (5-ALA) are valuable adjuncts in the treatment of malignant brain tumors. Although their concomitant use was previously contraindicated due to photosensitivity concerns, a 2022 regulatory revision permitted their combined application. Transient postoperative hyperintensity on diffusion-weighted imaging (DWI) at the irradiation site serves as a biomarker for PDT effects, but the radiological and clinical impact of combining 5-ALA and TS remains unclarified. To compare and evaluate postoperative DWI findings and clinical outcomes in patients undergoing TS-PDT with or without concomitant 5-ALA-guided PDD. **Methods**: This retrospective study analyzed 34 patients with recurrent primary malignant brain tumors who underwent TS-PDT between January 2019 and November 2025. Patients were divided into a TS alone group (*n* = 19) and a TS + 5-ALA group (*n* = 15). Postoperative DWI hyperintensity thickness and minimum apparent diffusion coefficient (ADC) values at the laser irradiation site were measured. Adverse events including photosensitivity were also compared. **Results**: No significant differences were observed between the TS alone and TS + 5-ALA groups in DWI hyperintensity thickness (3.71 mm vs. 3.90 mm; *p* = 0.703 or median ADC values (601.0 × 10^−6^ mm^2^/s vs. 527.0 × 10^−6^ mm^2^/s; *p* = 0.205). Furthermore, there were no significant differences in the incidence of photosensitivity and liver enzyme elevation. **Conclusions**: Concomitant use of 5-ALA-PDD and TS-PDT can be used without worsening DWI findings at the PD laser irradiation site.

## Simple Summary

Surgical treatment of malignant brain tumors in Japan is often combined with photodynamic diagnosis (PDD) or photodynamic therapy (PDT), which involve the administration of light-activated agents that help visualize tumor boundaries (5-aminolevulinic acid) or destroy residual tumor cells (talaporfin sodium). Because both agents increase photosensitivity, their combination was previously avoided due to concerns about adverse effects. We are the first to compare postoperative brain reactions using diffusion-weighted imaging at the PD laser irradiation site between patients undergoing PDT alone and those undergoing combined PDD-PDT. Significant differences were not observed, indicating the feasibility of the PDD-PDT combination.

## 1. Introduction

Talaporfin sodium (TS) is a photosensitizer approved exclusively in Japan [1,2]. Since 2013, photodynamic therapy (PDT) with TS has been authorized as an intraoperative local adjuvant treatment for primary malignant brain tumors in Japan [3]. Clinical reports indicate that this method achieves effective local control of residual invasive cells within the resection cavity and is associated with favorable treatment outcomes [3,4]. Conversely, photodynamic diagnosis (PDD) serves as a surgical assistance technique that enhances the gross total resection (GTR) rate [5,6]. In Japan, 5-aminolevulinic acid (5-ALA) was approved in 2013 as an intraoperative diagnostic agent for malignant gliomas and is used exclusively for PDD [7]. Since both agents are photosensitizers that induce photosensitivity, their concomitant use was previously contraindicated. However, a revision of the package insert in 2022 allowed their concomitant use under limited conditions [8].

Early postoperative magnetic resonance imaging (MRI) after TS-PDT frequently reveals a transient diffusion-weighted imaging (DWI) hyperintense area at the photodynamic (PD) laser irradiation site. This finding has been reported as a useful biomarker reflecting the local effects of PDT [9,10]. The combined use of 5-ALA and TS has not yet become widely adopted nationwide, and the changes in postoperative DWI hyperintensity thickness and apparent diffusion coefficient (ADC) values associated with this combination have not yet been fully investigated.

Accordingly, the purpose of this study was to compare and evaluate postoperative DWI findings at the PD laser irradiation site in patients undergoing TS-PDT, focusing on the presence or absence of concomitant 5-ALA-guided PDD.

## 2. Materials and Methods

### 2.1. Patients and Study Design

This retrospective historically controlled cohort study was conducted at the University of Tsukuba Hospital (Ibaraki, Japan). The study included consecutive patients aged 18 years or older who underwent resection surgery combined with PDT using TS for recurrent primary malignant brain tumors at our hospital between January 2019 and November 2025. Before February 2023, our institutional policy was to perform TS-PDT alone; thereafter, we transitioned to a policy of combining TS-PDT with 5-ALA-guided PDD. Patients were divided into two groups based on this policy change for comparative analysis: the TS alone group and the TS + 5-ALA group. Because TS-PDT was not performed for newly diagnosed cases before February 2023, we excluded newly diagnosed cases.

Data on the following factors were collected from medical records: age, sex, Karnofsky performance status (KPS), and tumor laterality. GTR was defined as no residual lesions within the dura mater on the first postoperative MRI. Photosensitivity was considered positive if skin reactions, such as erythema, edema, or blisters, were observed using a sun sensitivity test 7–10 days after surgery. Elevated aspartate aminotransferase (AST) or alanine aminotransferase (ALT) was defined as an increase in AST or ALT to grade 2 or higher according to the Common Terminology Criteria for Adverse Events (CTCAE) v5.0 [11] in blood tests within 7 days postoperatively. Pathological diagnoses were also recorded.

### 2.2. Surgical Techniques, PDT, and PDD

5-ALA (20 mg/kg) was administered orally 2 to 4 h before surgery, and TS (40 mg/m^2^) was administered intravenously 22 to 26 h before surgery, in accordance with the package insert instructions. All surgical procedures were performed by the surgical team (all are board-certified neurosurgeons with at least 10 years of experience) using an M530 OH6 microscope (Leica Microsystems, Wetzlar, Germany) with or without 5-ALA fluorescence guidance. A 664 nm semiconductor PD laser (Panasonic Healthcare Co., Ltd., Tokyo, Japan) was applied to the resection cavity as specified in the package insert (150 mW/cm^2^, 27 J/cm^2^).

### 2.3. Postoperative MRI Assessment

To evaluate imaging changes after PD laser irradiation, we used the first postoperative DWI MRI using a 1.5–3-tesla system (Ingenia Ambition X, Ingenia, and MR7700, Philips Healthcare, Best, The Netherlands). In principle, MRI was performed within 48 h postoperatively for malignant gliomas and on postoperative day 7 for meningiomas. DWI was acquired with a single-shot spin-echo echo-planar imaging (SS-SE-EPI) sequence (b-values of 0 and 1000 s/mm^2^; slice thickness 5.0 mm; interslice gap 0.5 mm; 25 slices). For Ingenia Ambition X, other parameters were: TR 4000 ms; TE 75 ms; flip angle 90°; FOV 230 mm; matrix 128 × 256; SENSE 2.0; voxel size 1.80 × 1.29 × 5.00 mm. For Ingenia and MR7700, the parameters were as follows: TR 6250 ms; TE 68–81 ms; flip angle 90°; FOV 230 mm; matrix 160 × 256; SENSE 3.0; voxel size 1.44 × 1.46 × 5.00 mm.

**Figure 1:**
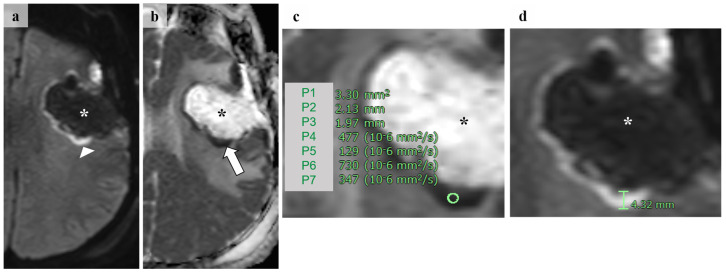
DWI hyperintensity thickness and ADC value measurement. A representative postoperative MRI demonstrates an area of restricted diffusion at the intraoperative PD laser irradiation site (indicated by the arrowhead and arrow) around the resection cavity (asterisk). (**a**) Diffusion-weighted image (DWI). (**b**) Apparent diffusion coefficient (ADC) map. (**c**) Enlarged view of the ADC map, with the circle indicating the region of interest (ROI). The measured parameters (P1–7) for the ROI are listed from top to bottom: area, major axis, minor axis, mean, standard deviation, maximum, and minimum. (**d**) Enlarged view of the DWI, with the line indicating the thickness of DWI hyperintensity.

DWI hyperintensity thickness and corresponding ADC values were measured by one of the authors (T.I.) according to previous studies [9,10]. First, we identified the location within the intraoperative PD laser irradiation site with the lowest ADC value, defined a circular region of interest (ROI) with a 2 mm diameter centered on that point, and calculated the mean ADC value. Next, within the region containing that site, we measured the maximum thickness of the DWI hyperintensity perpendicular to the resected cavity wall (Figure 1). Since the penetration depth of PD lasers is generally considered to be up to 5 mm [12,13], and the thickness of DWI hyperintensity within the irradiation field is typically 7 mm or less postoperatively [9,10], this study defined cases in which the thickness of the DWI hyperintensity exceeded 7 mm as ischemic changes (infarction) and excluded them from the analysis.

### 2.4. Ethical Considerations

This study was approved by the University of Tsukuba Clinical Research Review Board (approval number: R01-202) and performed in accordance with the principles of the Declaration of Helsinki and the Ethical Guidelines for Medical and Biological Research Involving Human Subjects in Japan. We conducted this observational study based solely on existing data held by our institution, on an opt-out basis in accordance with national research regulations and the instructions of the institutional ethics committee, and the requirement for informed consent was waived. All clinical information was handled anonymously in accordance with the Japanese National Act on the Protection of Personal Information.

### 2.5. Statistical Analysis

Continuous and categorical variables were analyzed using the Mann–Whitney U test and Fisher’s exact test, respectively. Continuous variables are reported as the median and interquartile range. Based on the achieved sample sizes of our cohort, a sensitivity power analysis was conducted with α = 0.05 and a power of 0.80. All statistical analyses were performed with EZR version 1.70 (R commander version 2.9–5), a graphical user interface for R version 4.5.2 (The R Foundation for Statistical Computing, Vienna, Austria) [14]. Probability values below 0.05 were considered significant.

## 3. Results

### 3.1. Patient Background

**Figure 2:**
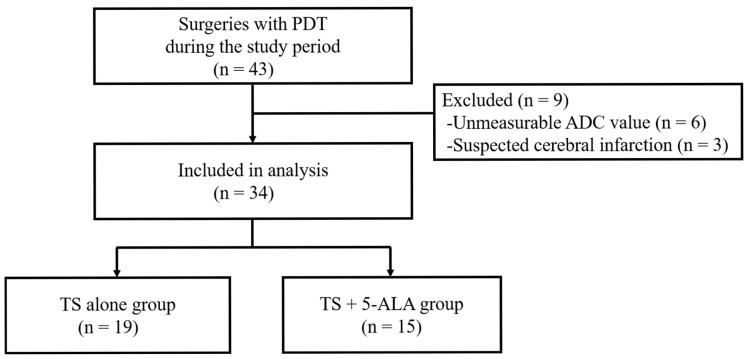
Study flowchart.

We performed 43 resection surgeries with TS-PDT during the study period; we excluded 6 patients with unmeasurable ADC values and 3 patients with ischemic changes exceeding 7 mm on postoperative MRI, leaving 34 patients for final analysis (Figure 2). The cohort comprised 19 patients in the TS alone group and 15 in the TS + 5-ALA group. The patient characteristics are shown in Table 1. No significant differences were found between the two groups.

### 3.2. DWI Hyperintensity Thickness and ADC Value

Postoperative MRI demonstrated that the median ADC values were 601.0 × 10^−6^ mm^2^/s for the TS alone group and 527.0 × 10^−6^ mm^2^/s for the TS + 5-ALA group, with no significant difference observed between the groups (*p* = 0.205; Hodges-Lehmann estimated difference: 84.0; 95% confidence interval (CI): −58.0–216.0) (Table 2). When the analysis was limited to cases imaged at 3 tesla MRI within 48 h postoperatively (i.e., excluding meningiomas and cases evaluated at 1.5 tesla) for sensitivity analysis, the results remained similar (596.5 [444.0–678.0] × 10^−6^ mm^2^/s in the TS-alone group, *n* = 14; 508.0 [415.5–574.5] × 10^−6^ mm^2^/s in the TS + 5-ALA group, *n* = 11; *p* = 0.366). The thickness of DWI hyperintensity was 3.71 mm in the TS alone group and 3.90 mm in the TS + 5-ALA group, with no statistically significant difference (*p* = 0.703; Hodges-Lehmann estimated difference: 0.15; 95% CI: −0.67–1.05). The analysis after excluding meningiomas and cases evaluated at 1.5 Tesla revealed similar results (3.93 [3.24–5.18] mm in the TS-alone group, *n* = 14; 4.31 [3.73–4.60] mm in the TS + 5-ALA group, *n* = 11; *p* = 0.848).

We performed sensitivity power analyses (α = 0.05, power = 0.80) based on the achieved sample size (*n* = 19 in TS alone group and *n* = 15 in the TS + 5-ALA group). The minimal detectable differences in ADC value and DWI hyperintensity thickness between the two groups were 225–237 × 10^−6^ mm^2^/s (Cohen’s d = 0.98) and 1.22–1.29 mm (Cohen’s d = 0.98), respectively.

To minimize temporal confounding associated with long-term shifts in general perioperative care and diagnostic standards, we conducted a sensitivity analysis restricted to patients treated in close temporal proximity to the transition period (i.e., the latter half of the TS alone group vs. the early half of the TS + 5-ALA group). The results remained similar to the original analysis (ADC values: 633.5 [479.8–739.0] × 10^−6^ mm^2^/s in the TS-alone group, *n* = 10 vs. 550.0 [486.3–622.0] × 10^−6^ mm^2^/s in the TS + 5-ALA group, *n* = 8; *p* = 0.408) (DWI hyperintensity thickness: 3.40 [3.04–4.03] mm in the TS-alone group vs. 3.56 [2.91–4.13] mm in the TS + 5-ALA group; *p* = 0.897).

### 3.3. Clinical Outcomes

Although the difference was not significant, GTR was achieved in 66.7% of patients in the TS + 5-ALA group compared with 47.4% in the TS alone group (*p* = 0.314) (Table 3). Regarding adverse events, there were no significant differences between the groups in the incidence of photosensitivity (5.3% in the TS alone group versus 13.3% in the TS + 5-ALA group, *p* = 0.571; odds ratio (OR) = 2.69; 95% CI: 0.13–172.1) or AST/ALT elevation (10.5% in the TS alone group versus 6.7% in the TS + 5-ALA group, *p* = 1.000; OR = 0.62; 95% CI: 0.01–13.0).

Sensitivity power analyses revealed that the minimal detectable differences between the two groups were 42.54% for photosensitivity (baseline rate: 5.26%) and 46.57% for AST/ALT elevation (baseline rate: 10.53%).

## 4. Discussion

Since 2022, our institution has fully implemented a general policy of combining TS-PDT with 5-ALA-PDD to enhance resection rates. We reported that this combination can be implemented without major complications [8]. Nevertheless, since both drugs share similar photosensitizing mechanisms, concerns remain about a potential increase in adverse events, particularly photosensitivity, and the intensification of PDT-specific local tissue reactions [15]. In this study, clinical indicators, including photosensitivity and serum AST/ALT levels, as well as postoperative MRI findings, such as DWI hyperintensity thickness and ADC values, did not differ significantly between the TS alone and TS + 5-ALA groups.

The antitumor effects of PDT are mediated by three primary mechanisms: direct destruction of tumor cells through apoptosis and necrosis, damage to tumor vasculature, and activation of an immune response with local cytokine release [4,12,16,17]. PDT induces rapid necrosis and ischemic changes in tumor tissue and the adjacent vascular endothelium, leading to cellular edema and restricted water diffusion within the intra- and extracellular spaces [3,18,19]. These changes are thought to produce high signal intensity on DWI at the site of PD laser irradiation [9,10,16,20]. Furthermore, some researchers suggest that DWI hyperintensity following PDT reflects the tumor-suppressing effect of PDT [10]. In this study, both groups exhibited a thickness of just under 4 mm, which is slightly less than that reported in previous studies. This difference is likely attributable to variations in the MRI scanners or imaging conditions across different facilities [21,22].

The absence of significant differences in radiographic changes observed in this study may be attributed to the differing excitation wavelengths of the two agents. The excitation wavelength for TS in clinical applications is centered at 664 nm, while protoporphyrin IX (PpIX), a metabolite of 5-ALA, has its primary absorption peak for PDT between 630 and 635 nm [12,15,16,23]. According to the physical absorption spectrum, PpIX absorbs minimal light near 660 nm, suggesting a low risk of PpIX excitation by the PD laser irradiation used for TS-PDT [24,25]. In contrast, xenon light sources in surgical microscopes and surgical lights emit a broad range of visible wavelengths, which can excite both TS and PpIX [26,27]. Microscope lights, in particular, are extremely intense. The OH6 model used in this study is equipped with a 400-watt xenon lamp and is reported to potentially exceed 550,000 lx at close range (Leica M530 OH6 Technical Specifications, Leica Microsystems GmbH). However, significant difference in DWI findings was not observed between the two groups. A possible explanation for this outcome is that the resection cavity wall (the site of PD laser irradiation) was not exposed to white light under the microscope for a duration sufficient to deliver the excitation energy needed for cellular damage. Importantly, the observation that imaging findings indicative of worsening cellular damage (such as DWI hyperintensity and ADC values) did not worsen, even when 5-ALA was administered in combination with TS, is valuable for reducing psychological barriers to combination therapy.

Several limitations should be acknowledged. This investigation was conducted at a single center, used a retrospective design with a limited sample size, and included only recurrent cases. The treatment era differs between the groups, which can introduce confounding factors, such as proficiency in surgical techniques. Image analysis was performed in an open-label manner by a single author. Although significant differences in adverse events were not observed between the two groups based on imaging and clinical indicators, the sensitivity analyses showed that the study had sufficient power only to detect large differences between the groups. Notably, the small number of clinical adverse events limits the accuracy of assessment. Therefore, this study alone is insufficient to draw definitive conclusions regarding safety. Furthermore, hard endpoints such as overall survival were not evaluated, which precludes definitive conclusions regarding the clinical impact of combining PDT and PDD. Although this study found no difference in the GTR rate, achieving GTR through re-resection of recurrent glioblastoma is associated with improved prognosis [28,29,30]. Therefore, the strategy of combining PDT and PDD to enhance resection rates warrants further investigation.

## 5. Conclusions

This study compared cases of recurrent primary malignant brain tumors treated with TS-PDT with and without the addition of 5-ALA-PDD. The addition of 5-ALA did not result in any significant changes in the postoperative DWI hyperintensity thickness or ADC values, nor did it worsen clinical adverse events such as photosensitivity or elevated liver enzyme levels. Although this observational study of 34 cases has limited statistical power, the cohort suggests that using 5-ALA-guided PDD together with TS-PDT for recurrent primary malignant brain tumors may be feasible. Future prospective comparative trials are warranted.

## Figures and Tables

**Table 1:** Patient characteristics.

	Number (%) or Median [Interquartile Range]			*p* Value
	Total, *n* = 34	TS Alone Group, *n* = 19	TS + 5-ALA Group, *n* = 15	
Age	55.0 [50.0–67.0]	55.0 [50.5–67.5]	56.0 [48.5–67.0]	0.862
Sex, male	19 (55.9%)	8 (42.1%)	11 (73.3%)	0.091
Preoperative KPS	90 [70–90]	80 [70–90]	90 [70–90]	0.302
Tumor laterality, left	17 (50.0%)	9 (47.4%)	8 (53.3%)	1.000
Diagnosis				0.437
-Glioma, grade 4	25 (73.5%)	13 (68.4%)	12 (80.0%)	
-Glioma, grade 3	6 (17.6%)	3 (15.8%)	3 (20.0%)	
-Meningioma, grade 2/3	3 (8.8%)	3 (15.8%)	0 (0%)	
Magnetic field strength, 3 tesla	28 (82.4%)	17 (89.5%)	11 (73.3%)	0.370

5-ALA, 5-aminolevulinic acid; KPS, Karnofsky performance status; TS, talaporfin sodium.

**Table 2:** Postoperative DWI findings.

	Median [Interquartile Range]		*p* Value
	TS Alone Group, *n* = 19	TS + 5-ALA Group, *n* = 15	
ADC value (10^−6^ mm^2^/s)	601.0 [457.0–725.0]	527.0 [419.0–602.5]	0.205
DWI hyperintensity thickness (mm)	3.71 [3.16–5.16]	3.90 [3.22–4.54]	0.703

5-ALA, 5-aminolevulinic acid; ADC, apparent diffusion coefficient; DWI, diffusion-weighted image; TS, talaporfin sodium.

**Table 3:** Postoperative clinical findings.

	Number (%) or Median [Interquartile Range]		*p* Value
	TS Alone Group, *n* = 19	TS + 5-ALA Group, *n* = 15	
Gross total resection	9 (47.4%)	10 (66.7%)	0.314
Photosensitivity	1 (5.3%)	2 (13.3%)	0.571
Elevated AST or ALT	2 (10.5%)	1 (6.7%)	1.000
KPS at discharge	70 [60.0–80.0]	80 [65.0–90.0]	0.227

5-ALA, 5-aminolevulinic acid; ALT, alanine aminotransferase; AST, aspartate aminotransferase; KPS, Karnofsky performance status; TS, talaporfin sodium.

## Data Availability

Raw data were generated at the Department of Neurosurgery, University of Tsukuba. Derived data supporting the results of this study are available from the corresponding author (N.S.) on request.

## Abbreviations

The following abbreviations are used in this manuscript:

5-ALA	5-aminolevulinic acid;
ADC	apparent diffusion coefficient;
ALT	alanine aminotransferase;
AST	aspartate aminotransferase;
CI	confidence interval;
CTCAE	Common Terminology Criteria for Adverse Events;
DWI	diffusion-weighted imaging;
FOV	field of view;
GTR	gross total resection;
KPS	Karnofsky performance status;
MRI	magnetic resonance imaging;
OR	odds ratio;
PD	photodynamic;
PDD	photodynamic diagnosis;
PDT	photodynamic therapy;
PpIX	protoporphyrin IX;
ROI	region of interest;
SS-SE-EPI	single-shot spin-echo echo-planar imaging;
TS	talaporfin sodium;
vs.	versus
